# Negative impact of SARS-CoV-2 infection in acute coronary syndrome mortality in a Latin American cohort study

**DOI:** 10.3389/fmed.2022.959769

**Published:** 2022-09-21

**Authors:** Wenderval Borges Carvalho Junior, Neila Nunes Ferreia, Luciano de Moura Santos, Patrícia Brito de Almeida Borges, Cleandro Pires de Albuquerque, Laila Salmen Espindola, Otávio de Toledo Nóbrega, Ciro Martins Gomes, Licia Maria Henrique da Mota, Alexandre Anderson de Sousa Munhoz Soares

**Affiliations:** ^1^Graduate Program in Medical Sciences, Faculty of Medicine, University of Brasilia-UnB, Brazilia, Brazil; ^2^University Hospital of Brasilia, HUB-UnB-EBSERH, Brazilia, Brazil

**Keywords:** SARS-CoV-2, COVID-19, acute coronary syndrome, myocardial infarction, Brazil

## Abstract

**Purpose:**

COVID-19 infection has been associated with a high risk of complications and death among patients with acute coronary syndrome (ACS). However, there is little information on the simultaneous involvement in Latin American countries.

**Methods:**

In the period between May 2020 and February 2021, an observational, longitudinal, prospective cohort study with two parallel branches was conducted in private and public hospitals in Brasilia, Brazil, including patients with ACS with and without a positive SARS-CoV-2 test result during hospitalization.

**Results:**

A total of 149 patients with ACS were included (75 with COVID-19 and 74 controls). Patients with COVID-19 exhibited an average of 62 years of age, 57% men, 40% diabetics, 67% hypertensive, 48% had an ACS with ST-segment elevation, Killip I was predominant, a low Syntax Score in 72%, with an average Grace Score of 117, and a length of hospitalization of 43 days in average. The control branch was similar in clinical characteristics, except for a lower proportion of ST-segment elevation ACS (16%, *p* < 0.01) and a higher incidence of arrhythmias (8 vs. 20 %, *p* = 0.03). Using the Cox regression method of analysis of covariates collected in the study, it was identified that patients with COVID-19 had a risk of death 2.34 times higher than patients without COVID-19 (*p* = 0.049).

**Conclusion:**

In this study conducted in a Latin American capital, SARS-CoV-2 infection predicted a higher chance of death in patients admitted with ACS, which is a finding that reinforces the need for greater care when diseases develop in overlapping ways.

## Introduction

SARS-CoV-2 is the causative agent of a global pandemic, affecting more than 180 countries worldwide, with the epicenter in Wuhan, China. It can cause a plethora of symptoms ranging from dry cough, fever, and myalgia to acute respiratory failure, all of which characterize COVID-19 (1, 2).

The effect of COVID-19 on the cardiovascular system occurs *via* the ACE-2 receptor, which is not as abundant as in the pulmonary system but exists in the cardiovascular system to a lesser extent, in addition to the intestinal epithelium, vascular endothelium, and kidneys, which ultimately explains the diffuse nature of compromise caused by the virus (3, 4).

Myocardial injury, presenting as increased cardiac biomarkers, is correlated with clinical severity. As reported in a previous study (1), elevated troponin I above the reference limit was observed in 46% of patients who did not survive and in only 1% of those who survived. There are two troponin elevation patterns: a stabilized increase and a persistent increase in the days following hospitalization (5). The persistent increase in troponins implies a worse prognosis, explained by the associated increase in other inflammatory biomarkers, such as D-dimer, ferritin, interleukin-6, and lactate dehydrogenase, and occurs secondary to the hemophagocytic process rather than directly from an isolated myocardial injury.

There are also other forms of presentation of COVID-19 in the cardiovascular system in addition to the myocardial injury of ischemic etiology: acute viral myocarditis, stress cardiomyopathy, disseminated intravascular coagulation, inflammatory cytokine release, arrhythmias, and deep vein thrombosis. In addition to these cited forms, Ruan et al. (6) demonstrated that among 68 deaths among a series of 150 patients, 33% were due to fulminant myocarditis.

Age is another risk factor that impacts COVID-19 mortality. Yang et al. (7) reported that the impact of age with regard to COVID-19 manifests in the mortality rate of patients older than 80 years (14.8%) and younger than 70 years (4%). Possible explanations are that elderly individuals have more advanced cardiovascular diseases, have a faulty immune system, and have high levels of ACE-2 receptors leading to a greater predisposition to COVID-19.

Cardiovascular diseases are still the major cause of death in developing countries, such as Brazil, with acute coronary syndrome (ACS) representing a large proportion of the cases. In the context of the pandemic, most societies in the cardiovascular area established specific criteria for care and protocols to mitigate the impairment of care of patients with ACS, as well as to promote adequate conditions for health professionals to reduce the contagion of them and other hospitalized patients (8, 9).

With the unfolding of the COVID-19 pandemic, an additional challenge is imposed on the Brazilian and Latin American population as a whole, as this is a part of the world notoriously known as an endemic region for other infectious diseases such as Chagas Disease, dengue, and malaria resulting in a triple burden to the cardiovascular health services. This trifecta of diseases posed not only diagnostic and therapeutic challenges, but also additional challenges of mobilizing emergency care, navigating patient influx through the cardiovascular care system, and training teams in minimizing the spread of the virus.

## Objectives

The main objectives of this study were to evaluate mortality and length of hospital stay among patients who concomitantly exhibited two overlapping syndromes (ACS in patients known to have COVID-19) and to compare the results with those of the control group of patients with ACS without COVID-19 in the period from May 2020 to February 2021 during the first year of local dissemination of the COVID-19 pandemic in Latin America.

## Methods

### Study design

An observational, longitudinal, prospective study of cohorts with two parallel arms was conducted. The inclusion criteria were those who developed, during the COVID-19 pandemic period, episodes of ACS, with and without ST elevation on electrocardiography, in cardiology units, hemodynamic laboratories, and intensive care units of one public and two private hospitals in Brasilia (the capital of Brazil). Patients were recruited between May 2020 and February 2021 to meet the eligibility criteria for the study on the first day of admission. Due to the special difficulty of differentiation between acute or chronic myocardial injury of ischemic or other etiology, cases were only included in the study after coronary angiography confirmed the presence of at least one severe obstructive lesion (> or = 70% in any epicardial vessel) to minimize, but not exclude other etiologies of myocardial injury and the diagnosis of ACS was confirmed by at least two different cardiologists considering the Fourth Universal Definition of Myocardial Infarction consensus (10). Patients were enrolled between May 2020 and February 2021, consecutively, in the proportion of 1 to 1 cases of COVID-19 positive and negative, which formed the control group. Patients were thoroughly informed and consensually agreed to take place in this study during their hospitalization period. This study received approval from the Ethics Committee of the Faculty of Medicine of the University of Brasília—UnB.

All participants underwent RT-PCR (part of all recruiting hospitals protocols at admission or in case of symptoms) and serology for SARS-CoV-2 (specific test for the study conducted on the last day of hospitalization and/or 10th day of hospitalization, using serological kits approved and registered by local regulatory agencies) and were then allocated to the exposure (RT-PCR and/or IgM antibodies positive for SARS-CoV-2) or control (RT-PCR and IgM serology negative for SARS-CoV-2) groups.

First, the exposed group was established; subsequently, the control group was then identified and formed. When a control patient with ACS, though asymptomatic, tested positive for SARS-CoV-2 *via* a serology exam, the patient was then transferred to the exposed group, and a new case was sought to replace that patient in the control group. Other complications from the viral infection, such as secondary bacterial pneumonia or venous thromboembolism, were not considered exclusion criteria. The inclusion of all patients happened before the beginning of vaccination against COVID-19 started to take place in Brasilia, Brazil.

Demographic variables were collected from the medical records of the included patients: age; sex; clinical comorbidities such as hypertension, diabetes mellitus, chronic obstructive pulmonary disease, and previous cardiac arrhythmias or cardiac arrhythmias developed during hospitalization, defined by the absence of sinus rhythm. The data on cardiac arrhythmias were documented at admission and daily in a binary field in the medical record, so specific electrocardiograms were not acquired for the study. In addition, the following markers of severity of ACS were collected: the presence of ST-segment elevation, the Killip-Kimball clinical classification (11), clinical Grace Score (12), and Syntax score (13); the latter calculated a posteriori by an independent blinded examiner regarding the diagnosis of COVID-19.

After discharge, all patients were followed by the cardiology staff of the same hospital they were first admitted at least for one medical visit after discharge. The follow-up could be prolonged if the assisting physician judged that the patient still has complications or need further interventional treatment for the ACS. The vital status was collected until the last visit that the patient attended. Loss of follow-up was considered if the patient could not be reached to attend a medical visit ordered by the assisting cardiologist, but the researchers did not intervene in the decision to discharge each patient from the ambulatory follow-up.

### Statistics

The sample size was based on a *t*-test for independent samples, with alpha = 0.05 and beta = 0.2, to detect a significant difference between the mean length of hospital stay between the groups (primary outcome), with a moderate effect size (Cohen's *d* = 0.5). The sample size was calculated as *N* = 126 patients. The data are presented as ±SD for normally distributed data or median (interquartile range) for non-parametric data, in addition to absolute numbers (relative percentage in the group) for categorical variables. For the comparison of the groups, the Student, Mann–Whitney, and Fischer's exact *t*-tests were used, respectively, according to the type of variable to be compared. The mortality analysis consisted of fitting Cox regression models for the time in days until the death associated or not with COVID-19 adjusted for epidemiological and clinical covariates using the hazard ratio (HR) as an effective measure and the respective CI. The analysis was performed in two stages, bivariate and multiple, and both HRs and their respective 95% CIs were calculated. Initially, simple Cox regression models were fitted for each covariate. Those in which the *p*-value was less than 0.25 were included in the multiple Cox regression analysis. Subsequently, adjustments were made to these variables through a process of variable removal/inclusion. Only covariates with *p* < 0.05 remained in the final model. Subsequently, an independent variable of interest was included, whether or not COVID-19 was present, to verify the degree of association between COVID-19 and the time until death after adjustment for possible confounders. Finally, the hazard ratios (HRs) and their respective 95% CIs were calculated. Kaplan–Meier estimates of survival functions and log-rank test were subsequently applied to compare the two branches according to SARS-CoV-2 infection. A significant *p* < 0.05 was considered. The analyses were conducted by the SAS 9.4 software.

## Results

A total of 149 consecutive patients with a clinical diagnosis of ACS who met the study eligibility criteria were included, 75 in the exposed group and 74 in the control. Table 1 provides the clinical and epidemiological characteristics. For the patients with COVID-19, the mean age was 62 years, 45 % were men, 40% had diabetes, 57% were hypertensive, 72% had low SYNTAX scores, and 16% were classified as Killip class > I, with a mean GRACE score of 117 and length of hospital stay on average of 43 days. In the comparison between the groups, no significant differences were observed except for a higher proportion of ACS patients with ST-segment elevation in SARS-CoV-2 exposed (*p* = 0.001) and a lower incidence of arrhythmias (*p* = 0.03).

**Table 1:** Clinical and epidemiological characteristics of patients according to SARS-CoV-2 infection.

	**SARS-CoV-2**		***p***
	**Positive (75)**	**Negative (74)**	
Age, years	62 ± 13	65 ± 13	0,203
Male sex, *n*	52 (57)	40 (43)	0,055
Diabetes, *n*	30 (40)	26(35)	0,534
Hypertension, *n*	50 (67)	59 (80)	0,072
COPD, *n*	10 (13)	7 (9)	0,475
Cardiac arrythmias, *n*	6 (8)	15 (20)	**0,026**
ST-segment elevation, *n*	36(48)	12 (16)	**< 0,001**
Killip > 1, *n*	12 (16)	13(18)	0,768
Grace score, points	117 ± 39	114 ± 35	0,817
SYNTAX score of low severity, *n*	54 (72)	47(64)	0,757
Length of hospital stay, days	43 ± 60	34 ± 30	0,915

Continuous variables expressed as average ± standard deviation. Categorical variables expressed as number (%). n, number; COPD, chronic obstructive pulmonary disease. Bold values are considered statistically significant.

The overall mortality was 17.4% during follow-up, 17 (22.7%) patients died in the exposed group, and 9 (12.2%) patients died among controls (*p* = 0.091). All deaths occurred during the first hospitalization period; although there were cases of programmed and urgent rehospitalization during the ambulatory follow-up, they did not result in deaths. Loss of follow-up occurred in 12% of patients with concomitant COVID-19 and ACS and 8% in patients who presented initially only with an ACS. In the bivariate analysis, the occurrence or absence of COVID-19 was non-significantly associated with time until death (*p* = 0.0561). The covariates age, Killip classification, GRACE score, and chronic obstructive pulmonary disease (COPD) had a *p*-value < 0.25 and were also included in the multivariate model (Table 2). However, the final multivariate model showed that patients with COVID-19 had a 2.34 times higher risk of death than patients without COVID-19 (*p* = 0.0498). In addition, patients with a Killip classification between II and IV or with COPD had a higher risk of death than their counterparts: 3.02 (*p* = 0.0095) and 2.65 (*p* = 0.0317) times, respectively.

**Table 2:** Bivariate (gross HR) and multivariate (adjusted HR) Cox regression results to assess predictors of mortality in patients with acute coronary syndrome on hospital admission.

	**Gross HR**	Adjusted HR*
	**HR (95%CI), *p value***	**HR (95% CI), *p value***
Age ≥ 60 years	3.79 (1.13–12.72). **0.030**	
Male sex	1.47 (0.58–3.73). 0.411	
Diabetes	2.13 (0.84–5.39) 0.109	
Hypertension	1.35 (0.50–3.61) 0.5535	
COPD	2.94 (1.22–7.09) **0.017**	2.65 (1.09–6.45) **0.0317**
Cardiac arrhythmias	1.63 (0.61–4.37) 0.333	
Absence of ST–segment elevation	1.01 (0.43–2.36). 0.983	
Grace score ≥ 140	3.47 (1.54–7.84). **0.003**	
Killip > I	3.22 (1.41–7.38) **0.006**	3.02 (1.31–6.96) **0.0095**
COVID−19	2.29 (0.98–5.35) 0.056	2.34 (1.00 – 5.49) **0.0498**

^*^Only the covariates that presented *p* < 0.25 in the bivariate analysis and remained with *p* < 0.05 in the multivariate model were included. HR, hazard ratio; COPD, chronic obstructive pulmonary disease. Bold values are considered statistically significant.

**Figure 1:**
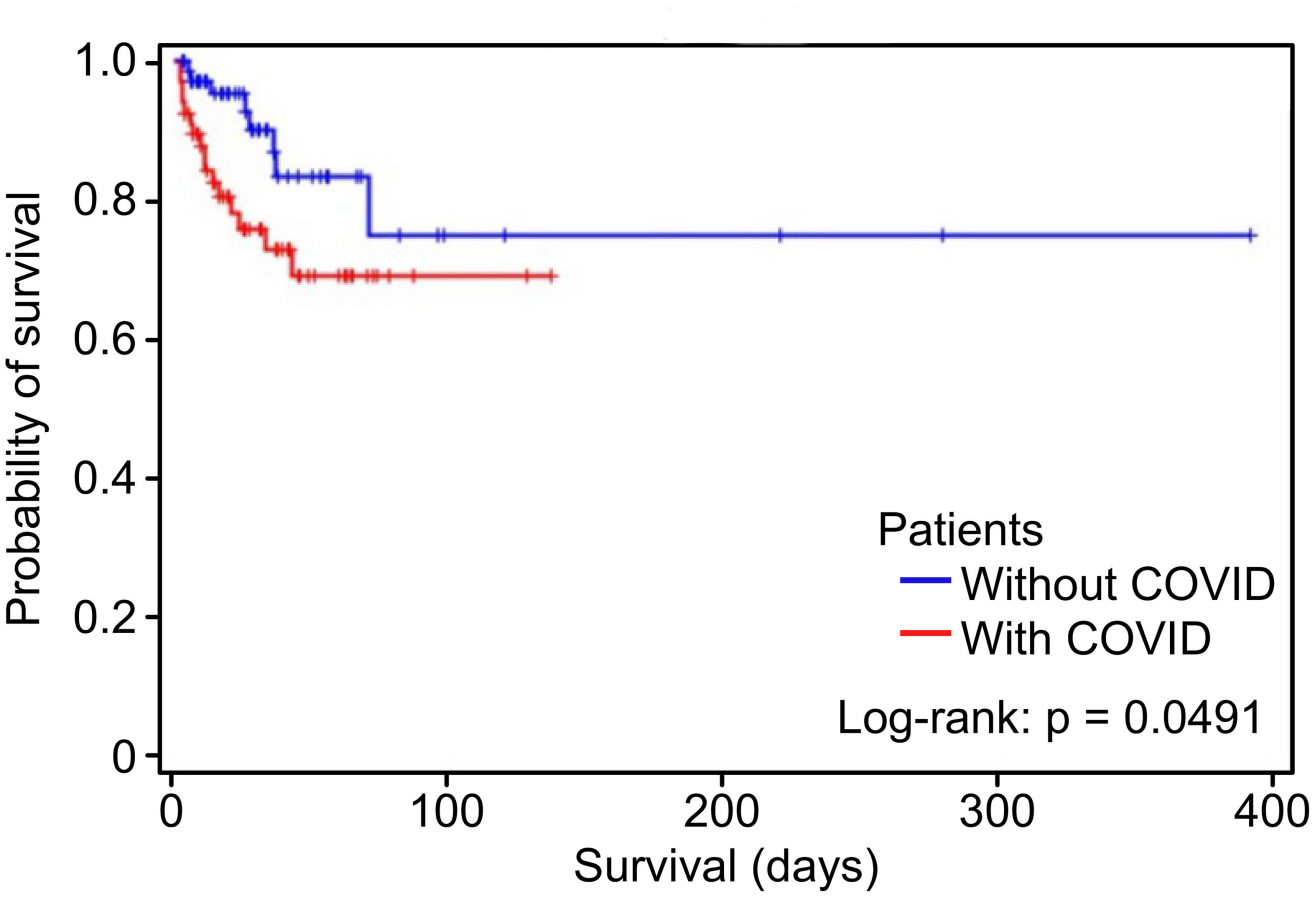
Kaplan–Meier curve for hospital death in the groups with the acute coronary syndrome with and without COVID-19.

Survival functions for the follow-up time in days for patients with and without COVID-19 were estimated by Kaplan–Meier analysis (Figure 1). The survival functions were compared using the log-rank test, and patients with SARS-CoV-2 had a worse prognosis (*p* = 0.0491).

## Discussion

In this sample of patients hospitalized for ACS in Latin American hospitals, exposure to SARS-CoV-2 was independently associated with lower survival, but it did not influence the length of stay in the hospital, which was heightened in both groups during the pandemic.

Viral infections, such as influenza, can be a triggering element of myocardial infarction. With COVID-19, this relationship is more significant. The analyses by Kwong et al. (14) showed that patients with acute respiratory infections have a higher risk of developing acute myocardial infarction (IR—incidence ratio 6.1, 95-CI: 3.9–9.5) after influenza infection than after non-influenza infections (IR −2.8, 95% CI: 1.2–6.2). However, an intense inflammatory response and hemodynamic changes can make plaques more susceptible to rupture (15). A previous study (16) indicated that having COVID-19 is an independent predictor of acute myocardial infarction and stroke in a given population in Europe. It is not known whether this is a direct effect of COVID-19 or a result of delayed treatment stemming from policies adopted to restrict movement, thus reducing access to hospitals dedicated to the care of cardiovascular problems (17).

One case series (18) included patients admitted with typical ECG abnormalities with ST-segment elevation, invasively stratified, but without evidence of atherosclerotic disease on coronary angiography. All patients tested positive for COVID-19. This indicates the enormous number of presentations of the disease and the difficulty of management and decision-making as the medical community was still scrambling to adjust to the restraint and complications of a global pandemic.

In the present study, the length of hospital stay for patients without and with COVID-19 in the context of ACS was not significantly different. Notably, our sample of patients was small compared to that in other studies (19). Importantly, during the initial stages of the pandemic, the differential diagnosis of ACS and COVID-19 was extremely challenging, and the fear of health professionals with regard to infection and the organizational logistics in decision-making within hospital services may have affected the length of stay. However, regarding the length of hospitalization, studies that report differences compared groups with data that were collected outside the context of the COVID-19 pandemic, and therefore, there was no negative control group for COVID-19 (20). In our study, there was a negative control, and all data were collected during the pandemic.

The Strategy of Registry of Acute Coronary Syndrome (ERICO) cohort that was conducted in a university public hospital in Brazil during the pre-COVID period showed a shorter length of stay with a median of 8 days and a lower mortality of 30% than in the present study, although patients' baseline characteristics were similar with a median of 62 years, 59% male, 40% with diabetes, and 77% with hypertension (21). However, this length of stay and mortality of ACS in Brazil can reach double of other developed countries in the world (22). It must be mentioned that public recruiting hospital was not performing cardiac surgeries during the first 8 months of the local dissemination of SARS-CoV-2, and in the whole public Brasilia health system, only urgent cardiac surgeries were being performed in most of 2020 and the beginning of 2022 which affect greatly the length of hospital stay of some study patients. When considering patients with COVID-19, the catheterization labs of the recruiting hospitals also accepted only urgent cases, and elective angioplasties were deferred to minimize staff contamination. Besides the COVID-19 pandemic, another explanation for our findings of the long length of stay and high mortality is the important regional differences in treatment of ACS in Brazil, with less use of demonstrably effective therapies in the Midwest region of Brazil, where Brasilia is situated, and in public hospitals (23).

In this study, mortality among those patients who exhibited the two concomitant syndromes was significant, corroborating the literature outside of Latin America (19, 20). The difficulty of diagnosis, atypical symptoms, the fear of infection in hospitals, and the delay in the diagnosis of ACS may also have contributed to worse outcomes for our patients. In addition, the direct and indirect actions of the virus through increased inflammatory activity are elements that may also have contributed to the worse clinical outcomes observed among our patients.

## Limitations of the study

This study was affected by the reduced number of ACS events at the peak of the pandemic, an effect that was observed worldwide, with a significant reduction (17) of more than 50% resulting from isolation policies and fear of infection. This phenomenon is believed to be a result of social distancing policies, which confined patients to their homes, as well as fear of contagion. So, the current widespread vaccination against SARS-CoV-2 must have affected the present results not just from the biological viewpoint of the interaction between COVID-19 and ACS but also from the social perspective. The door-to-balloon time was not evaluated in the study, the course of action taken after patient stratification was not determined, and patient follow-up was not standardized among the institutions involved in this study. As no data about COVID-19 and ACS interaction were available at the time of this study design to support sample size estimation and as we chose to evaluate a moderate effect size, this study is not able to discard the small effect size of COVID-19 in the length of stay. It also must be noted that our sample size calculation was based on the length of stay rather than mortality. Another possible cofounder in the survival analysis was the heterogeneous time of follow-up with outliers with a very long follow-up due to complications of surgeries and the need for repeated coronary interventions. Finally, as most deaths occurred after long hospitalization periods and several complications, the specific cause of each death lacked precision, so it is not reported in the present study.

## Conclusion

The results from this study indicate increased mortality among patients with ACS and COVID-19, a finding that corroborates the severity of the two diseases when they occur concomitantly in Latin American patients. The length of hospital stay was high during the pandemic period, but it did not differ significantly between patients with ACS with or without COVID-19.

## Data availability statement

The raw data supporting the conclusions of this article will be made available by the authors, without undue reservation.

## Ethics statement

The studies involving human participants were reviewed and approved by Comite de Ética em Pesquisa da Faculdade de Medicina da Universidade de Brasília (CEP/FM - UnB). The patients/participants provided their written informed consent to participate in this study.

## Author contributions

WJ, CA, LM, and AS contributed to conception and design of the study. NF and WJ organized the database. AS performed the statistical analysis. WJ and AS wrote the first draft of the manuscript. WJ, NF, LS, PB, CA, LE, ON, CG, and LM wrote sections of the manuscript. All authors contributed to manuscript revision, read, and approved the submitted version.

## Conflict of interest

The authors declare that the research was conducted in the absence of any commercial or financial relationships that could be construed as a potential conflict of interest.

## Publisher's note

All claims expressed in this article are solely those of the authors and do not necessarily represent those of their affiliated organizations, or those of the publisher, the editors and the reviewers. Any product that may be evaluated in this article, or claim that may be made by its manufacturer, is not guaranteed or endorsed by the publisher.
